# Synthesis, characterization, and evaluation of antibacterial activity of transition metal oxyde nanoparticles

**DOI:** 10.1007/s10856-021-06578-8

**Published:** 2021-08-18

**Authors:** Dielly Oliveira Morais, Alexandre Pancotti, Guilherme Sastre de Souza, Marielena Vogel Saivish, Alexandre Braoios, Marcos Lázaro Moreli, Mauro Vinícius de B. Souza, Vivaldo G. da Costa, Jiale Wang

**Affiliations:** 1 Universidade Federal de Jataí, Unidade Acadêmica Especial de Ciências Exatas, Rod. Br 364, km 168, Jataí, GO Brazil; 2 College of Science, Donghua University, Shanghai, 201620 China

## Abstract

Nanoparticles (NPs) have a wide range of applications in various areas. For health application, cytotoxicity tests are used to ensure its efficiency and safety. In this paper, ZnFe_2_O_4_, CoFe_2_O_4_, Zn_0.5_Co_0.5_Fe_2_O_4_ NPs were synthesized, characterized and their antibacterial properties were evaluated. The Sol-Gel method was used to synthesize the NPs. Their electronic and crystallographic structures were characterized by Fourier Transform Infrared Spectroscopy Analysis (FTIR), X-ray fluorescence (XRF), X-Ray Diffraction (XRD), and Transmission Electron Microscopy (TEM). To perform the antibacterial evaluation, ferrites were dispersed through nanoemulsion to prevent the crystals from accumulating together. Then the evaluation was performed through microdilution in a 96-well plate and diffusion in agar disc in contact with 3 different strains of *Staphylococcus aureus* and *Escherichia coli*. It demonstrated that the Sol-Gel method was efficient to synthesize NPs with suitable sizes for health application. All synthesized NPs showed the inhibition of bacterias with different concentrations used.

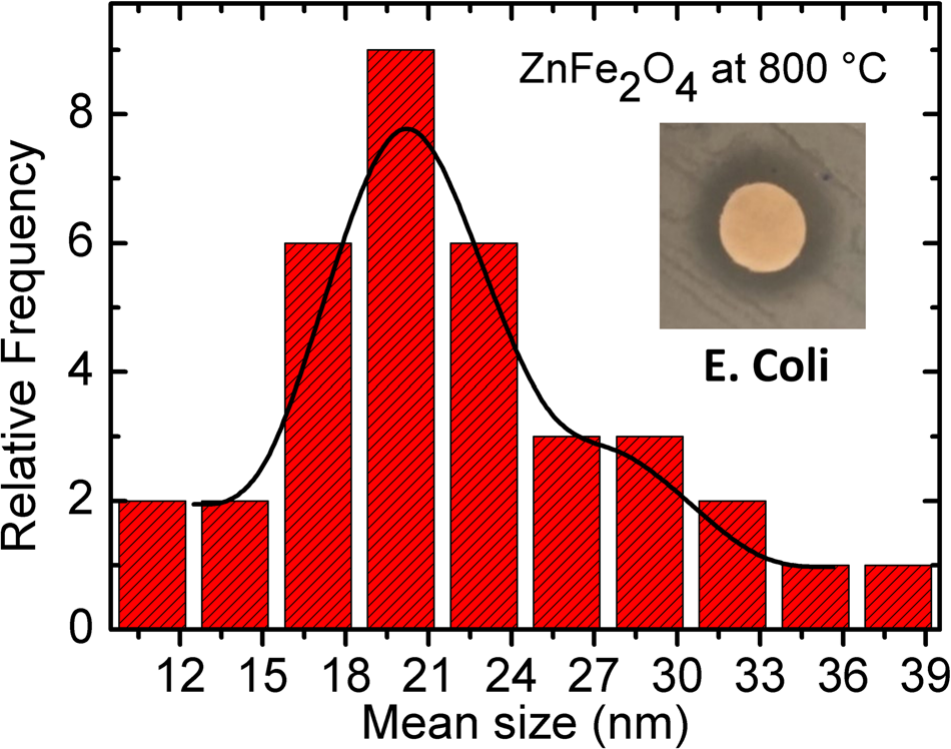

## Introduction

Nanotechnology is commonly known as a multidisciplinary science, involving the physical, chemical, and biological areas, where important applications are widely observed, such as in the industrial sector and in medicine [1]. The biological use of nanoscale materials, also known as nanoparticles (NPs), can be studied both in in vitro and in vivo [2, 3]. Various applications of NPs are related to the optical properties, size, shape, surface aspects, etc [4]. Because of their small size, unique physical–chemical characteristics have been observed [5]. Some NPs present magnetic characters that are composed of transition metals oxides, presenting the formula of MFe_2_O_4_, where M is a divalent metal, such as cobalt (CoFe_2_O_4_) and zinc (ZnFe_2_O_4_) [6–8]. Ferrites can be synthesized by multiple methods, i.e., solid-state reaction [9], polymeric precursors [10], thermal decomposition [11], hydrothermal [12], etc. The polymeric precursor method involves a sol–gel process, which starts with precursors in the liquid state, followed by the formation of the sol phase, which is a colloidal suspension. For the gel phase, organic precursors that have a chelating function are usually used, which may be citrate, gelatin, etc. And then the final product is calcined to form stable and homogeneous crystalline structures [10].

The incessant search for new antimicrobial drugs is due to the large number of microorganisms resistant to conventional antimicrobials [13], where NPs are considered as one of the alternatives. The activities provided by metal ions in biological environments have stimulated the research and development of these compounds as therapeutic agents in the treatment of infectious diseases [14]. Ag NPs accumulated in bacterial membrane can cause perforations in the membrane and result in the death of cell [15]. Ferrite NPs have an important role against Gram-positive and Gram-negative bacteria, suggesting that they can be used as antibacterial agents [16]. Ferrites NPs have efficient antibacterial properties against different bacterias.

Some properties of NPs can influence nanotoxicity. Lima et al. [17] demonstrated that the smaller the size, the greater the effect of cell uptake and toxicity. The charge, composition, and morphology are important factors related to the interaction of these materials with cellular components. Other factors such as physical–chemical characteristics, crystalline structure, solubility, and agglomeration are related to the mechanisms of action that cause toxicity [18, 19].

## Experimental

### Materials and methods

Metal salts of iron nitrate (Fe(NO_3_)_3_·9H_2_O), cobalt nitrate (Co(NO_3_)_2_·6H_2_O), zinc nitrate (Zn(NO_3_)_2_·6H_2_O), colorless gelatin, 5% citric acid, and 35% hydrogen peroxide (H_2_O_2_) (Sigma-Aldrich) were used. Mineral oil, tween 80 (Polysorbate 80), and propylene glycol were used for dispersion. In antibacterial tests, Mueller–Hinton (MH) broth and agar were used. And in the cytotoxic tests, MEM medium + 10% fetal bovine serum (FBS) + gentamicin (50 μg/mL), penicillin (100 IU/mL), amphotericin B (5 μg/mL), MTT, and DMSO were used.

### Preparation of ZnFe_2_O_4_ and CoFe_2_O_4_ NPs

The ZnFe_2_O_4_ and CoFe_2_O_4_ ferrite NPs were synthesized by using the sol–gel method, with a molar ratio of 1:2 (Co:Fe or Zn:Fe). To obtain the cobalt ferrite, 20.655 g of Fe(NO_3_)_3_·9H_2_O and 7.44 g of Co(NO_3_)_2_·6H_2_O were used. To obtain zinc ferrite, 20.655 g Fe(NO_3_)_3_·9H_2_O and 7.40 g of Zn(NO_3_)_2_·6H_2_O were weighed. Each salt was added, separately, in 90 mL of distilled water. In another 4 containers, 10.341 g of colorless gelatin was added in 90 ml of distilled water. The metal salts and gelatine were kept under constant stirring and heating at 40 °C for 40 min. After complete dissolution, gelatin and each nitrate were mixed separately, keeping under constant stirring and heating. And then cobalt nitrate was mixed with the solution. Finally, the solution was placed in a drying and sterilization oven at a temperature of 100 °C for 24 h to evaporate the water. The dry samples formed the xerogel and they were macerated until the formation of a homogeneous powder. The powders were calcined in a tubular oven at 250 and 800 °C for 4 h, with a heating rate maintained at 4 °C/min. Finally, the removal of residual organic matter was carried out by chemical treatment with 35% H_2_O_2_. The samples were washed with distilled water, followed by centrifugation, to separate the supernatant during synthesis. In the end, the samples were dried at 100 °C for 24 h [17].

### Preparation of NP Zn_0.5_Co_0.5_Fe_2_O_4_

Co^2+^ substituted zinc ferrite Zn_0.5_Co_0.5_Fe_2_O_4_ NPs was synthesized via polymeric precursor with 5% wt citric acid. The molar ratio of Zn:Co:Fe was 0.5:0.5:2, where 16.97 g Fe(NO_3_)_3_·9H_2_O, 3.05 g Co(NO_3_)_2_·6H_2_O, and 3.12 g Zn(NO_3_)_2_·6H_2_O were used, respectively. Each kind of salt was dissolved individually in a beaker with citric acid and kept under stirring at 70 °C for 40 min. And then they were mixed in a container and placed in an oven, drying at 100 °C for 12 h [20].

### Characterization studies

In order to characterize the structures of ferrites, Fourier transform infrared (FTIR) spectroscopy and X-ray fluorescence (XRF) were used. To perform the FTIR, the sample was placed in a JASCO FTIR-4100 sample holder. Data acquisition was performed in the range of 400–4000 cm^−1^ with a step of 0.01 cm^−1^. XRF was carried out on a Shimadzu model EDX-720. The measurements were performed in the range of 0–50 keV with a step of 0.01 keV. The experiments were performed at a pressure of 2.0 × 10^−8^ mbar. The XPS spectra were collected using a conventional Al K_α_ X-rays source with photon energy of 1486.6 eV. A VSW HA100 electron analyzer was used with 44 eV pass energy and 0.1 eV step. The base pressure in the analysis chamber was less than 5.0 × 10^−9^ mbar. The binding energy (BE) scale was ajusted using the C 1*s* line at 284.6 eV as a reference. A 10° takeoff angle was used to increase the surface sensitivity of the core-level peaks. The data were analyzed using the Winspec software. Shirley backgrounds were subtracted from the experimental data results.

XRD was performed with a Rigaku X-ray Diffractometer. The samples were analyzed using the diffractometer with the following parameters: voltage of 40 kV, current of 20 mA, low incidence angle of 10°, *θ*–2*θ* mode scan, range of 20–70°, scanning rate of 0.5°/min, and Cu K_α_ (*λ* = 1.541 Å) as source.

TEM was performed in a JEOL transmission electron microscope (JEM-2100) equipped with Thermoscientific EDS. The analyzed samples were prepared by adding 1 mg of the powder in an Eppendorf, where ethanol was used as a dispersing solvent and was placed in an ultrasound bath for 15 min. After dispersion, 250 µL of each sample was placed on the copper grid and rested on filter paper for ethanol evaporation. Then it was fitted in the microscope sample holder. The acceleration voltage used was 200.0 kV.

### Dispersion of ferrites

Nanoemulsion composed by mineral oil, tween 80, propylene glycol, and water were used in the proportion of 1:1:2:6 to form a stable dispersion of ferrites in an aqueous medium. Each component was sterilized by autoclaving, filtration, and ultraviolet radiation.

### Antibacterial tests

To perform the antibacterial tests, two methods were used: minimum inhibitory concentration (MIC) and disk diffusion on agar. Both tests were performed on three different strains of two species of bacteria: Gram-positive, *Staphylococcus aureus* ATCC (American Type Culture Collection) 25923, 29213, and 43300, as well as Gram-negative, *Escherichia coli* ATCC 25922, 51446, and 35218. Each strain was diluted in a saline solution, which was at the MacFarland scale 0.5, ~1.5 × 10^8^ UFC/mL [21].

The MIC method was performed to determine the lowest concentration capable of inhibiting the proliferation of the tested microorganisms. Thus, the NPs and strains were diluted in the following concentrations: 1 mg/mL, 0.5 mg/mL and 0.25 mg/mL. The tests were performed using 96-well plates with a “flat” bottom and in triplicate for each concentration of ferrite in MH broth. Three different types of inhibition controls were carried out. The positive inhibition controls were composed by Penicillin G Potassium at 10,000 UI/mL, and streptomycin sulfate at 10 mg/mL. The negative control was realized without the addition of microorganism. The microplates were incubated in an oven at 37 °C for 72 h. The microplate spectrophotometer was used to read the optical density, using the 630 nm primary filter, in the periods of 24, 48, and 72 h [22].

The disk diffusion test was realized according to the Kirby–Bauer Method. The inoculum of the microorganism was seeded over the entire surface of MH agar in a Petri disc. Then the NPs of 2 mg/mL were deposited on the agar surface. The plates were incubated in an oven at 37 °C for 24 h. The results were analyzed according to the presence or absence of a growth inhibition halo around the discs [20, 23–25].

### Determination of cell viability

The cytotoxicity of NPs was assessed by MTT assay. Briefly, Vero cells at a density of 1 × 10^5^ cells/mL were seeded in a flat-bottomed 96-well microtiter plate (Kasvi-Brazil) and were incubated for 24 h at 37 °C and 5% CO_2_, supplemented with 10% heat-inactivated FBS (Invitrogen, USA), 2 mM l-glutamine (Merck, Germany), and 100 U/ml penicillin and 100 μg/ml streptomycin sulfate (Sigma-Aldrich, USA). A range of concentrations from 62.5 to 1000 μg/ml of NPs was prepared using the cell culture medium DMEM and was added to the plate in triplicate. After 72 h, the treatments were removed and 100 μL of MTT reagent of concentration from 0.5 mg/mL in DMEM was added to each well and incubated for a further 2 h. The medium was then removed and 100 μL of DMSO solution was added to the wells. Finally, the plate was read at 550 nm by a microplate reader.

## Results and discussion

### FTIR

**Fig. 1:**
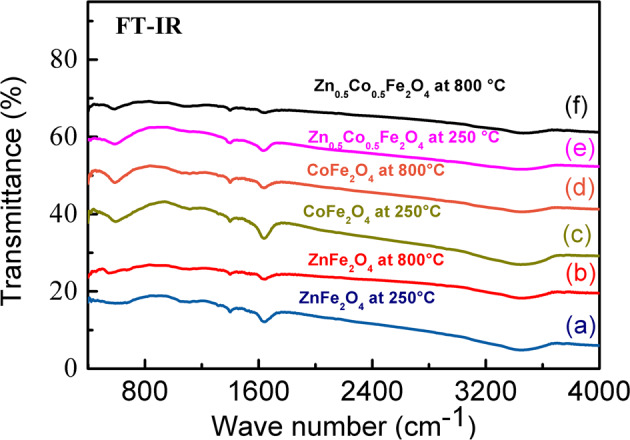
Typical FTIR spectrum of calcinated nanoparticles. FTIR spectroscopy of **a**, **b** ZnFe_2_O_4_, **c**, **d** CoFe_2_O_4_, and **e**, **f** Zn_0.5_Co_0.5_Fe_2_O_4_ calcined at 250 and 800 °C

Figure 1 exhibits the typical FTIR spectrum of ZnFe_2_O_4_, CoFe_2_O_4_, and Zn_0.5_Co_0.5_Fe_2_O_4_ NPs calcined in two different temperatures, which exhibits various well-defined peaks. The spectrum a (b) shows the absorption peaks for ZnFe_2_O_4_ calcined at 250 °C (800 °C). The well-defined peaks at 423 cm^−1^ (417 cm^−1^), 590 cm^−1^ (563 cm^−1^), 1098 cm^−1^(1107 cm^−1^), 1401 cm^−1^ (1398 cm^−1^), 1645 cm^−1^ (1636 cm^−1^), and 3448 cm^−1^ (3463 cm^−1^) were associated with the chemical bonds between the atoms of Fe–O, Zn–O, C=O, C–H, O–H, and O–H, respectively [26].

The spectrum c (d) shows the absorption peaks for CoFe_2_O_4_ calcined at 250 °C (800 °C). The peaks at 416 cm^−1^ (420 cm^−1^), 592 cm^−1^ (587 cm^−1^), 1120 cm−^1^(1085 cm^−1^), 1398 cm^−1^ (1401 cm^−^^1^), 1638 cm^−1^ (1628 cm^−1^), and 3461 cm^−1^ (3442 cm^−1^) were associated with the chemical bonds between the atoms of Fe–O, Co–O or Fe–O, C=O, C–H, O–H, and O–H, respectively [5, 27, 28].

The spectrum (f) shows the absorption peaks for Zn_0.5_Co_0.5_Fe_2_O_4_ NPs calcined at 250 °C (800 °C). The peaks at 412 cm^−1^ (418 cm^−1^), 583 cm^−1^ (575 cm^−1^), 1090 cm^−1^(1086 cm^−1^), 1403 cm^−1^ (1395 cm^−1^), 1635 cm^−1^ (1643 cm^−1^), and 3437 cm^−1^ (3465 cm^−1^) were associated with the chemical bonds between the atoms of Fe–O, Fe–O or Zn–O or Co–O, C=O, OH, OH, and OH, respectively. The first two peaks were attributed to the metal–oxygen bonds (Fe–O) in the tetrahedral sites, and the second peak was associated to the metal–oxygen bonds (Fe–O, Zn-O, and Co-O) in the octahedral sites. This is the typical characteristic of the reverse spinel and normal spinel for Co^2+^ substituted zinc ferrite Zn_0.5_Co_0.5_Fe_2_O_4_, magnetic NPs [27, 29–31].

### XRF

**Fig. 2:**
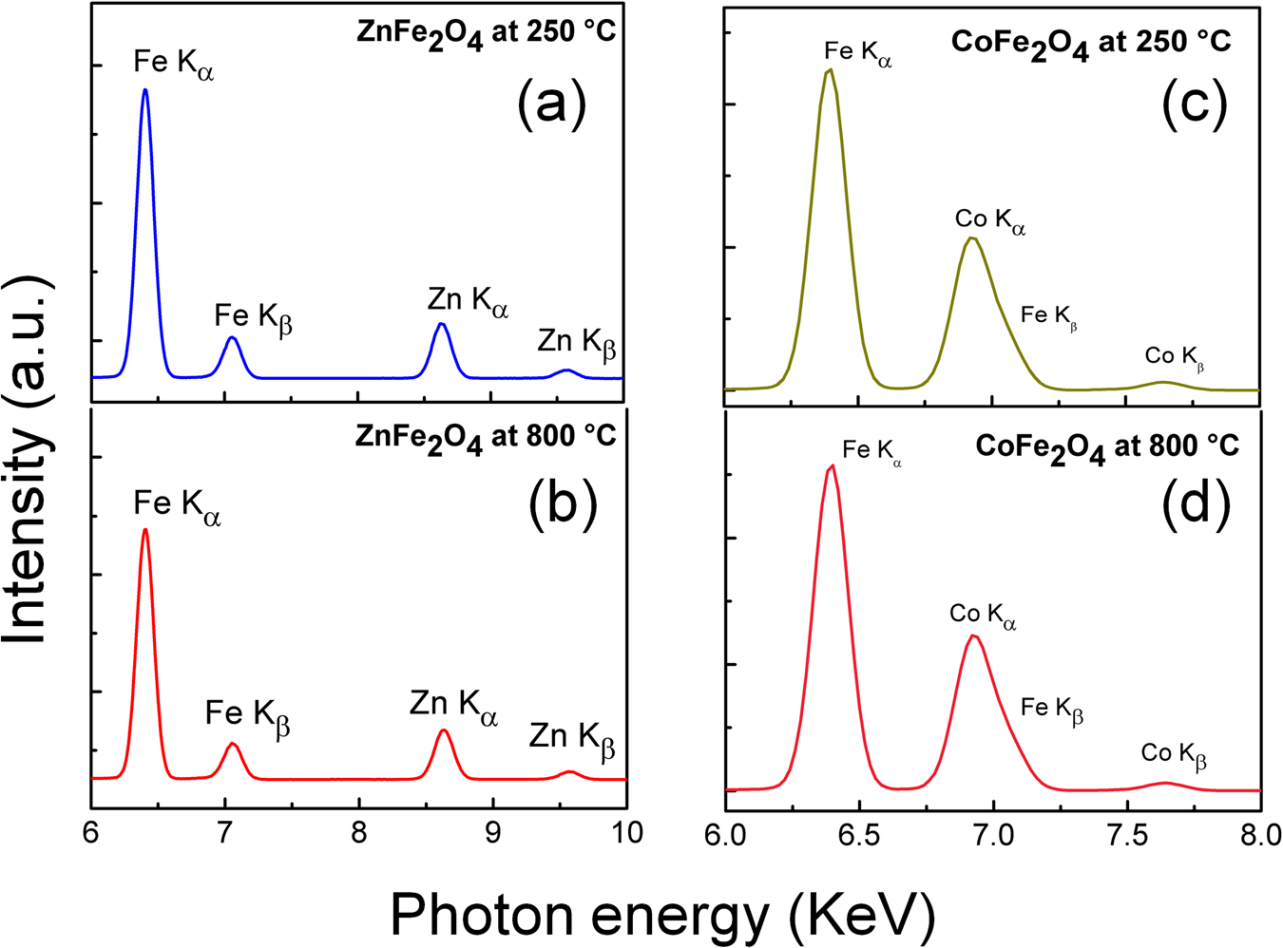
XRF spectra for the **a**, **b** ZnFe_2_O_4_ and **c**, **d** CoFe_2_O_4_ NPs calcined at 250 °C and 800 °C

Figure 2 shows the XRF spectra for the ZnFe_2_O_4_ and CoFe_2_O_4_ NPs calcined at different temperatures. In the Fig. 2a, b, the components present at 6.2 keV and 7.1 keV correspond to K_α_ and K_β_ lines of Fe atom. The components present at 8.6 and 9.5 keV are due to K_α_ and K_β_ lines of Zn atom. In Fig. 2c, d, the components present at 6.9 keV corresponds to K_α_ and K_β_ line of Co atom. The average chemical composition obtained for ZnFe_2_O_4_ is 65.5%-wt (66.4%-wt) Fe, 29.8%-wt (30.6%-wt) Zn for ZnFe_2_O_4_ calcined at 250 °C (800 °C). Meanwhile, the average chemical composition obtained for CoFe_2_O_4_ are 72.0%-wt (72.1%-wt) Fe, 26.5%-wt (26.8%-wt) Co for CoFe_2_O_4_ calcined at 250 °C (800 °C).

### XPS

**Fig. 3:**
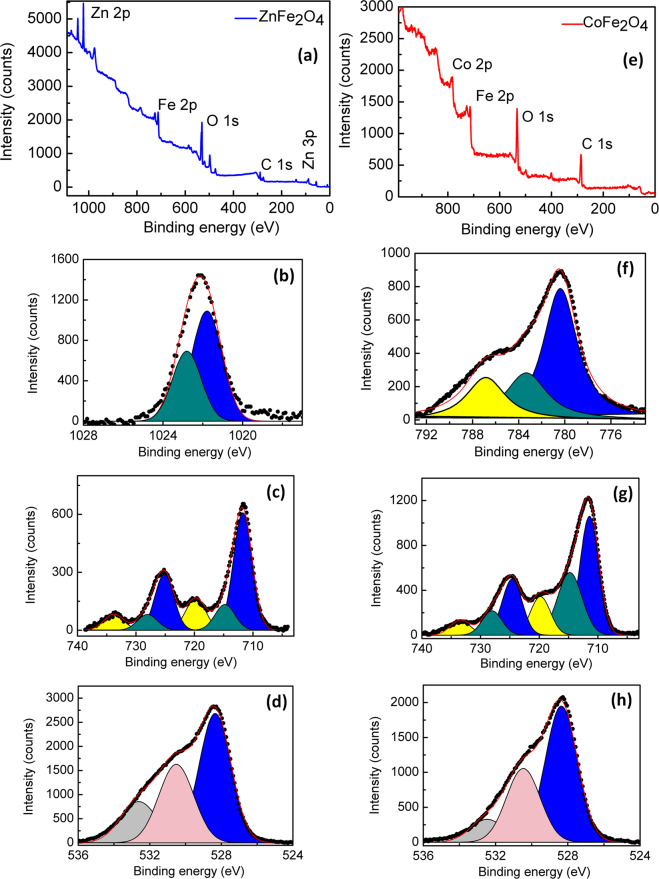
Survey XPS spectra for the **a** ZnFe_2_O_4_ and **e** CoFe_2_O_4_ NPs calcined at 800 °C. HRXPS for Zn ferrite nanoparticles calcined at 800 °C for: **b** Zn 2*p*; **c** Fe 2*p*; **d** O1*s*. HRXPS for Co ferrite nanoparticles calcined at 800 °C for: **f** Co 2*p*; **g** Fe 2*p*; **h** O1*s*

Wide-energy range XPS (survey) spectra were collected for assessing the cleanliness state of the NPs calcined at 800 °C are shown in Fig. 3. The atomic ratios determined by XPS are consistent with the CoFe_2_O_4_ and ZnFe_2_O_4_ NPs stoichiometry. There had been a significant amount of carbon at the surface, which was reduced by the calcination. The C 1*s* peak at 284.6 eV was used as a reference BE for calibration.

Figure 3b shows the measured HRXPS (high-resolution XPS) spectra of zinc ferrite NPs. Deconvolution of the Zn 2*p*_3/2_ peak was performed in the Zn 2*p* spectra as shown in Fig. 3b. The peak at 1021.4 eV was associated to Zn^2+^ occupying tetrahedral sites and the peak at 1022.6 eV was associated to Zn^2+^ occupying octahedral sites in zinc ferrite [32].

Figure 3c shows the Fe 2*p* core-level of ZnFe_2_O_4_ NPs calcined at 800 °C. The XPS spectrum has three components. The BE of 711.7 eV (Fe 2*p*_3/2_) and 725.1 eV (Fe 2*p*_1/2_) was associated to Fe^3+^ ions in octahedral site, with corresponding satellite peaks at 719.8 eV (Fe 2*p*_3/2_) and 733.8 eV (Fe 2*p*_1/2_), respectively. The BE of 714.8 eV and 728.0 eV, was related to Fe^3+^ ions in tetrahedral sites in Zn ferrite NPs [32].

Figure 3d shows the XPS for O 1*s* core-level of ZnFe_2_O_4_ NPs calcined at 800 °C. This spectrum is formed by three components presents at 528.3, 530.5, and 532.6 eV. The component present at 528.3 eV was associated to oxygen of ZnFe_2_O_4_ NPs and the components at 530.5 eV and 532.6 were associates to carbonate or hydroxyl groups chemically bound to surface cations of NPs [33–35].

Figure 3f shows the XPS spectra of Co 2*p*_3/2_ core-level calcined at 800 °C, which has three components with BE of 780.3, 783.3, and 787.3, and 783.3 eV were associated to Co^2+^ ions in the octahedral and tetrahedral sites [36, 37]. The peak at 787.3 eV was associated to the shake-up satellite peak of Co 2*p*_3/2_ main line [36].

Figure 3g shows the Fe 2*p* core-level of CoFe_2_O_4_ NPs calcined at 800 °C. The XPS spectrum has 3 components. The BE of 711.4 eV (Fe 2*p*_3/2_) and 724.6 eV (Fe 2*p*_1/2_) refers to the Fe^3+^ ions in octahedral site, with corresponding satellite peaks at 719.8 eV (Fe 2*p*_3/2_) and 733.4 eV (Fe 2*p*_1/2_), respectively. The BE of 714.8 eV and 728.0 eV, was associated to Fe^3+^ ions in tetrahedral sites.

The asymmetrical complex peak of the O 1*s* XPS spectrum (Fig. 3h) can be decomposed into three components, with BE of 528.3, 530.5, and 532.6 eV. Similar to the ZnFe_2_O_4_ NPs mention previously, the component present at 528.3 eV was related to oxygen of bulk structure and the components at 530.5 and 532.6 eV were associates to carbonate or hydroxyl groups chemically bound to surface cations of NPs [32, 38]. These XPS results are in perfect agreement with the XRD measurements.

### XRD

**Fig. 4:**
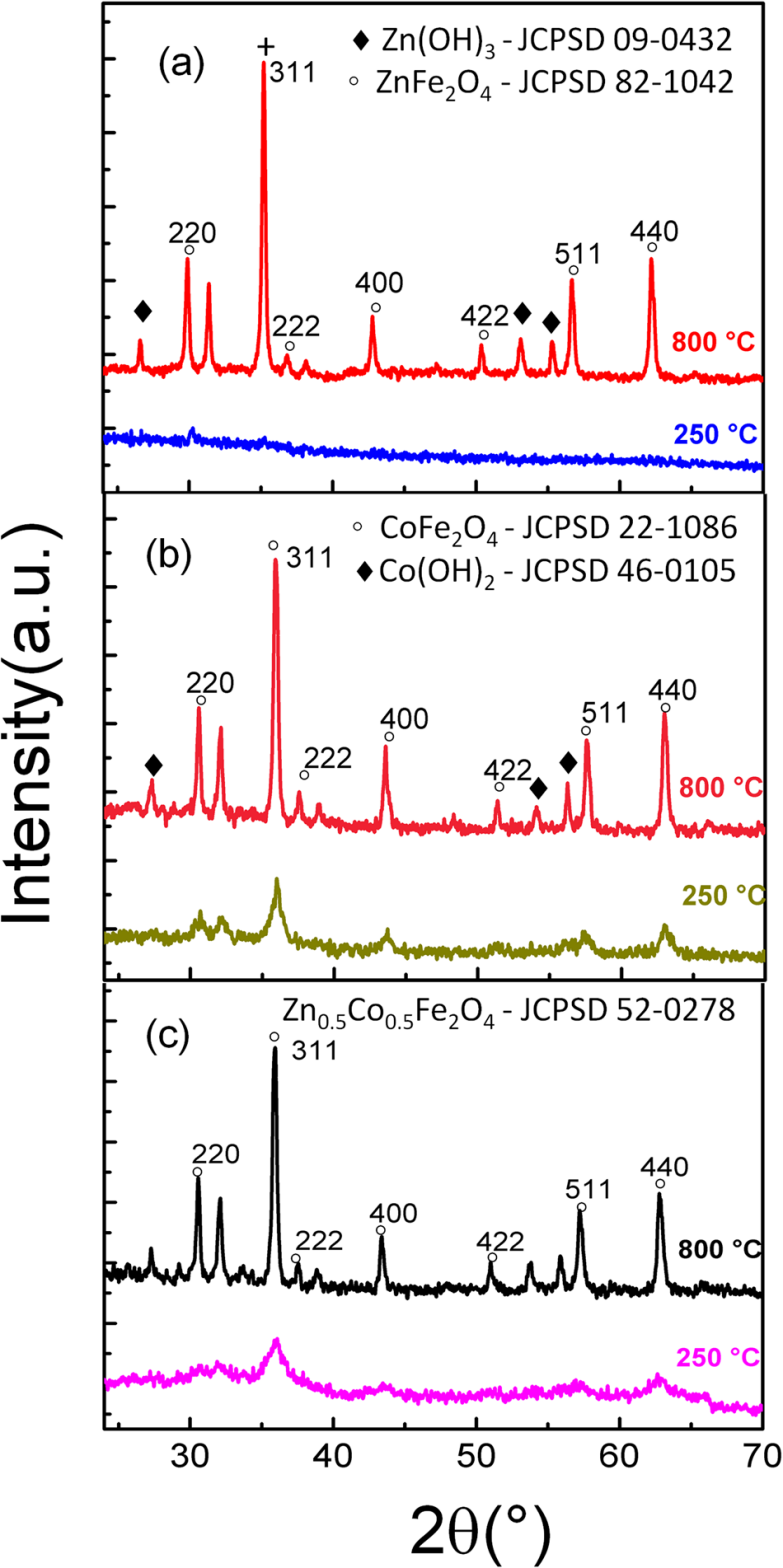
X-ray diffraction diffractograms for the ZnFe_2_O_4_ (**a**), CoFe_2_O_4_ (**b**) and Zn_0.5_Co_0.5_Fe_2_O_4_ (**c**) NPs calcinated at 250 and 800 °C

Figure 4a shows the XRD diffractograms of ZnFe_2_O_4_ NPs calcined at 250 and 800 °C. There were no diffraction peaks in the angular range acquired for the ZnFe_2_O_4_ calcined at 250 °C, demonstrating its amorphous characteristic. For the ZnFe_2_O_4_ NPs calcined at 800 °C, the peaks at 30°, 35°, 37°, 43°, 52°, 57°, and 62° were associated to (220), (311), (222), (400), (422), (511) and (440) facet, respectively.

Figure 4b shows the XRD diffractograms of CoFe_2_O_4_ NPs calcined at 250 °C and 800. For the sample calcined at 250 °C, the diffraction peaks at 32°, 36°, 43°, 52°, 57°, and 63° were associated to (220), (311), (400), (422), (511), and (440) facet, respectively. For the sample calcined at 800 °C, the diffraction peaks at 32°, 36°, 37°, 43°, 52°, 57°, and 63° were associated to (220), (311), (222), (400), (422), (511), and (440) facet, respectively. The change of the width in diffraction peaks suggested that NPs calcined at high temperatures have a larger size, as shown in the Table 1.

**Table 1:** Crystallite sizes (nm), where the 2*θ*, were obtained from the XRD diffractograms

Crystallite size - Scherrer equation (nm)							
Bragg angle (2*θ*)	Miller indice	Ferrites					
		ZnFe_2_O_4_ at 250 °C	ZnFe_2_O_4_ at 800 °C	CoFe_2_O_4_ at 250 °C	CoFe_2_O_4_ at 800 °C	Zn_0.5_Co_0.5_Fe_2_O_4_ at 250 °C	Zn_0.5_Co_0.5_Fe_2_O_4_ at 800 °C
30°	220	–	32.2	14.4	33.9	–	34.7
36°	311	–	35.9	17.1	25.4	9.3	29.6
37°	222	–	27.2	–	35.9	–	30.1
43°	400	–	41.3	26.5	45.9	–	34.8
52°	422	–	43.8	–	42.0	–	38.9
57°	511	–	41.1	25.2	38.8	–	38.2
62°	440	–	45.7	22.3	44.7	13.6	44.2

We have used the constant *k* = 0.91 and *λ* = 1.54 Å in the Scherrer Equation

Figure 4c shows the XRD diffractograms of Zn_0.5_Co_0.5_Fe_2_O_4_ NPs calcined at 250 and 800 °C. The diffraction peaks at 36°, 43°, 57°, and 63° corresponds to (311), (400), (511), and (440) facet, respectively. For the sample calcined at 800 °C, the diffraction peaks at 30°, 36°, 37°, 43°, 52°, 57°, and 63° were associated to (220), (311), (222), (400), (422), (511), and (440) facet, respectively. The change of the width in diffraction peaks also suggested that NPs calcined at high temperatures have a larger size, as shown in Table 1.

Table 1 shows the crystallites sizes for the synthesized NPs. The crystallites sizes were determined by Scherrer equation.

Table 1 shows that the crystallite sizes changed after the high-temperature calcination [35, 38] and showed that higher calcination temperatures produce higher intense peaks, increased grain size, and better crystallinity. These authors obtained 19 and 86 nm of cristallite size for zinc ferrite non-calcined and calcined for a longer time. Lima et al. [17] showed that the size of the NP has a great influence on cytotoxicity, where Co NPs produced using the sol-gel method, have greater cytotoxic power when low calcination temperatures are performed. Figueiredo et al. [39], measured the cristallites size equal to 12, 10, and 10 nm for zinc, cobalt, and mixed ferrites synthesized using the combustion reaction technique, without undergoing by calcination treatment.

### TEM

**Fig. 5:**
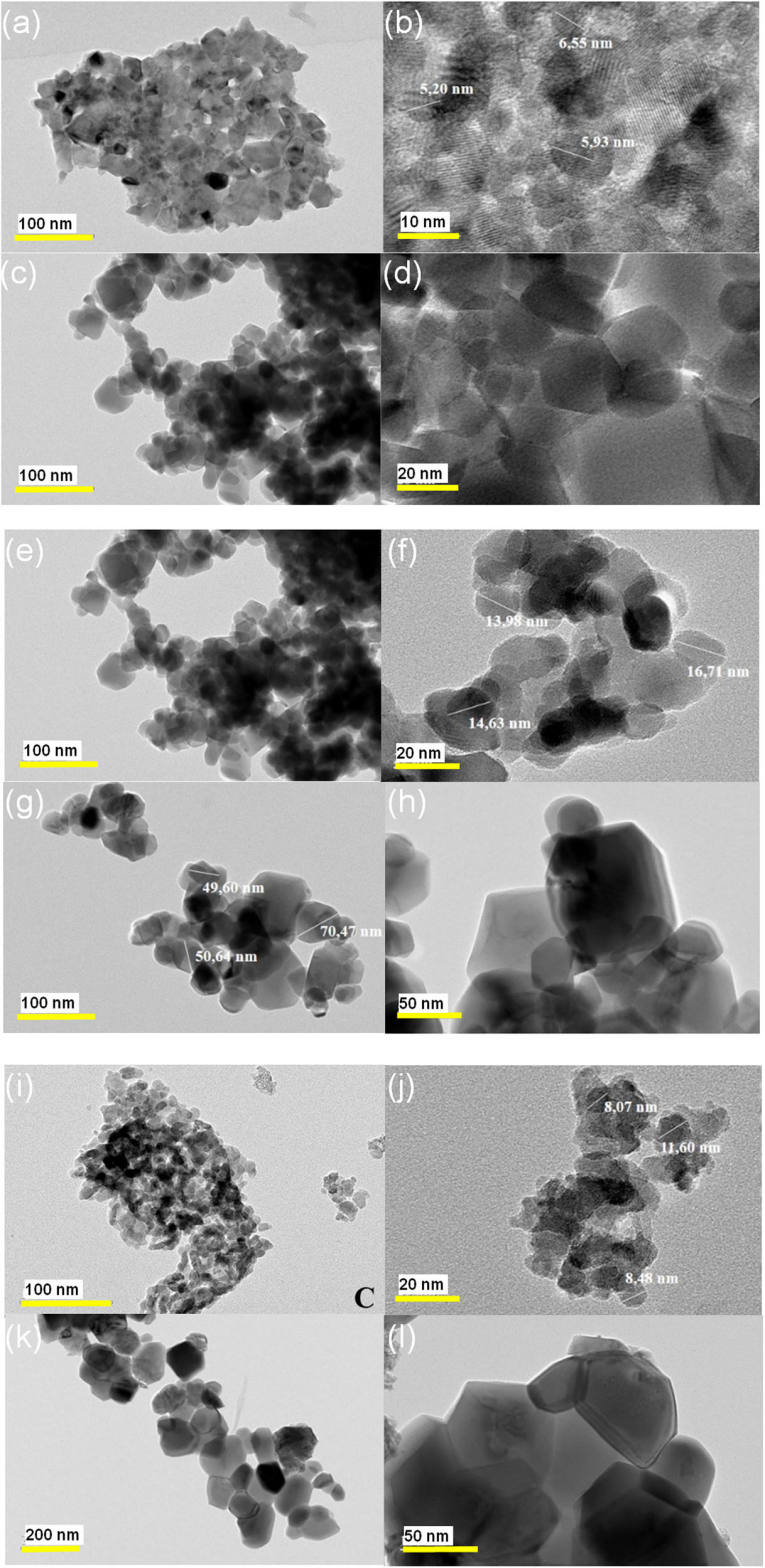
TEM images for ZnFe_2_O_4_, CoFe_2_O_4_, and Zn_0.5_Co_0.5_Fe_2_O_4_ NPs after calcination at 250 and 800 °C. ZnFe_2_O_4_ NPs after calcination at **a**, **b** 250 °C and 800 °C **c**, **d**. CoFe_2_O_4_ NPs after calcination at 250 °C (**e**, **f**) and 800 °C (**g**, **h**), and Zn_0.5_Co_0.5_Fe_2_O_4_ NPs after calcination at (**i**, **j**) 250 °C and 800 °C (**k**, **l**)

**Fig. 6:**
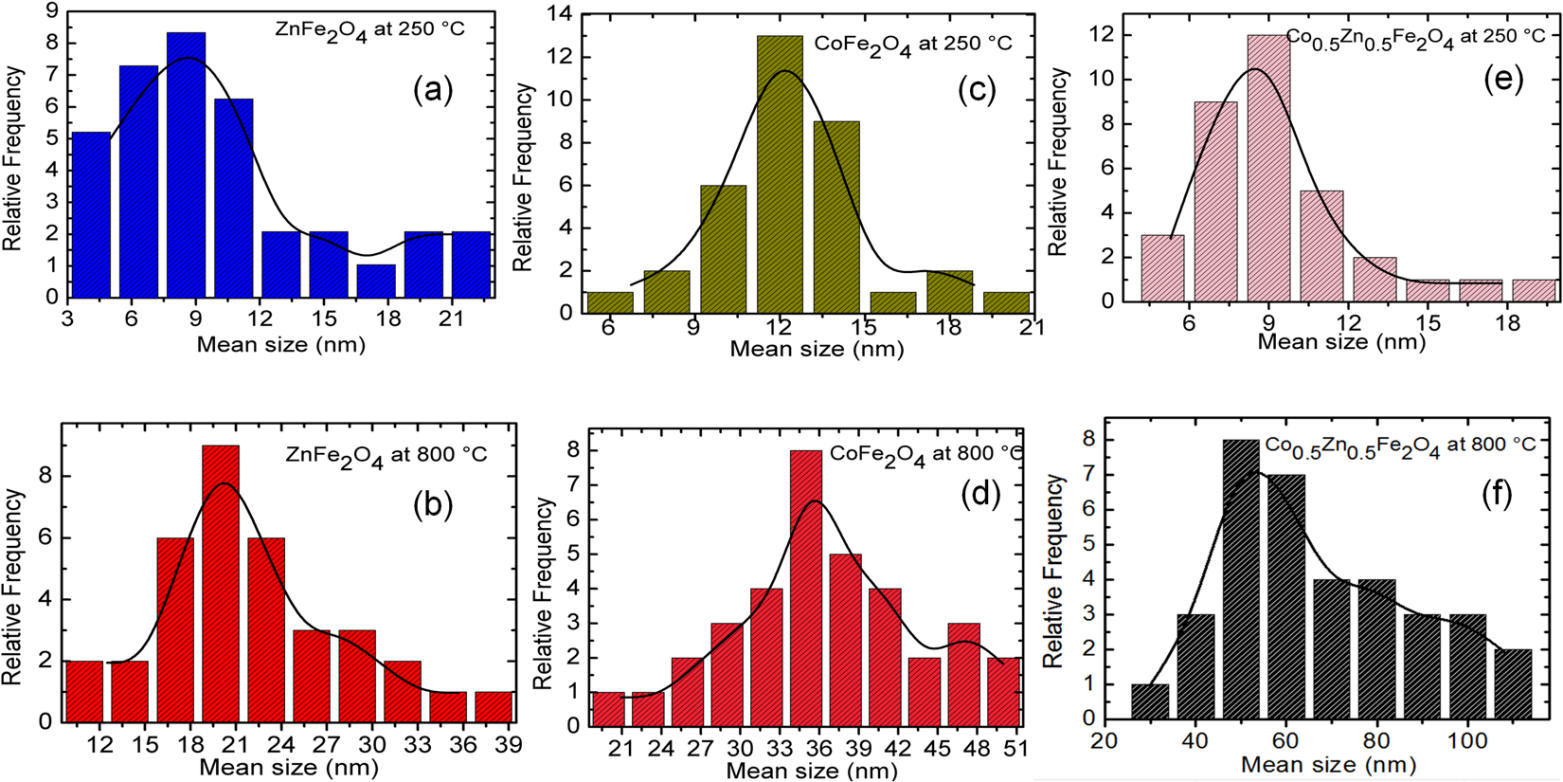
Histogram for ZnFe_2_O_4_, CoFe_2_O_4_, and Zn_0.5_Co_0.5_Fe_2_O_4_ NPs after calcination at 250 and 800 °C. ZnFe_2_O_4_ NPs after calcination at **a** 250 °C and 800 °C (**b**). CoFe_2_O_4_ NPs after calcination at **c** 250 °C and 800 °(**d**), and Zn_0.5_Co_0.5_Fe_2_O_4_ NPs after calcination at (**e**) 250 °C and 800 °C (**f**)

Figure 5 shows the TEM images of the ZnFe_2_O_4_, CoFe_2_O_4_, and Zn_0.5_Co_0.5_Fe_2_O_4_ NPs calcined at different temperatures. Their corresponding size histograms are depicted in Fig. 6, which were elaborated from the frequency sizes from the measurement of NPs by using Image J software. To obtain the histogram a total of *N* = 50 different particles sizes were used. Subsequently, a particle size histogram was mounted using the Sturges method [40]. The width (W) was obtained from the relation: W = (Dmax − Dmin)/*k*, where *k* = 1 + 3.322 log(N). The histogram is fairly well modeled by a log-normal distribution, as shown in Fig. 6 for all the NPs.

ZnFe_2_O_4_ sample calcined at 250 °C (Fig. 5a) showed characteristics of amorphous material, with an average size of 10.4 nm (Fig. 5b). For CoFe_2_O_4_ NPs calcined at 250 °C shown in Fig. 5e were observed the formation of partial amorphous structures. The same behavior was observed in the XRD measurements. These TEM images indicate that the CoFe_2_O_4_ and Zn_0.5_Co_0.5_Fe_2_O_4_ NPs calcined at 250 °C were relatively uniform, which were around 12.4 (Fig. 5f) and 9.2 nm (Fig. 5j) in diameter, respectively. The TEM images of ZnFe_2_O_4_, CoFe_2_O_4_, and Zn_0.5_Co_0.5_Fe_2_O_4_ NPs that were calcined at 800 °C are shown in Fig. 5c, g, k respectively. Here, the NPs sizes were larger compared to that calcined at 250 °C, which were around 21.9 (Fig. 5d), 36.7 (Fig. 5h), and 31.4 nm (Fig. 5l) for ZnFe_2_O_4_, CoFe_2_O_4_, and Zn_0.5_Co_0.5_Fe_2_O_4_ NPs, respectively.

### Antibacterial tests

**Fig. 7:**
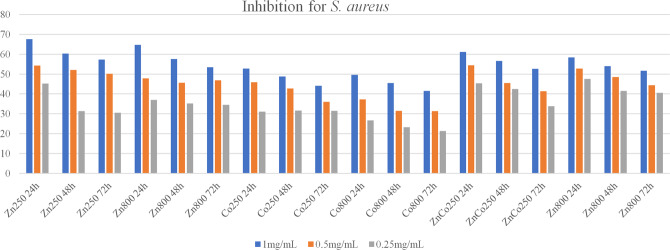
Percentage of growth inhibition of *S. aureus* strains (*n* = 3) presented by the tested concentrations of zinc, cobalt, and mixed ferrites dispersed by nanoemulsion, after incubation for 24, 48, and 72 h at 37 °C

**Fig. 8:**
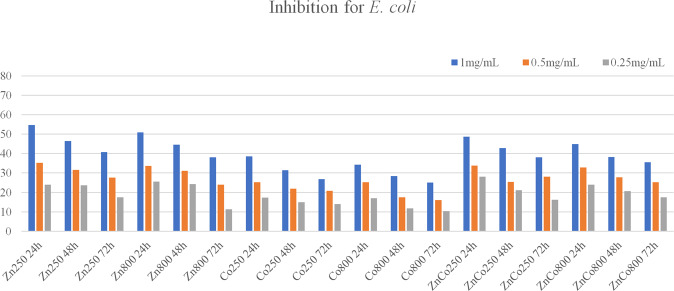
Percentage of growth inhibition of *E. coli* strains (*n* = 3) presented by the tested concentrations of zinc, cobalt, and mixed ferrites dispersed by nanoemulsion, after incubation for 24, 48, and 72 h at 37 °C

Inhibition tests using the MIC method showed, for all ferrites, that the highest inhibitory concentration is greater than 1 mg/mL. Thus, in Figs. 7 and 8, show the results with the concentrations of 1 mg/mL, 0.5 mg/mL, and 0.25 mg/mL according to the percentage of inhibition obtained using inhibition for the strains of *S. aureus* (*n* = 3) and *E. coli* (*n* = 3). This percentage was compared to the positive control for inhibition, which was a known antibiotic, and the negative control, which was the medium without the presence of inhibition. According to Fig. 7, the averages of inhibitions in the three incubation times are expressed for the ferrites calcined at 250 °C (800 °C) of ZnFe_2_O_4_, CoFe_2_O_4_, and Zn_0.5_Co_0.5_Fe_2_O_4_ in contact with the strains of *S. aureus* (*n* = 3). For ZnFe_2_O_4_ ferrite calcined at 250 °C they were 67.5%, 60.3%, and 57.2%, for 24 h, 48 h and 72 h, respectively. And the averages for ZnFe_2_O_4_ calcined at 800 °C were 64.7%, 57.5%, and 53.5%, respectively, for the three incubation times. For the CoFe_2_O_4_ calcined at 250 °C ferrite, the growth inhibition averages obtained, in the incubation times equal to 24, 48, and 72 h, were equal to 52.7%, 48.8%, and 44.1%, respectively. And for Co Fe_2_O_4_ calcined at 800 °C ferrite they were 49.6%, 45.5%, and 41.5%. For the Zn_0.5_Co_0.5_Fe_2_O_4_ ferrite calcined at 250 °C, the mean values of inhibition were obtained, in the incubation times 24, 48, and 72 h, equal to 61.2%, 56.6%, and 52.7%, respectively. And for the Zn_0.5_Co_0.5_Fe_2_O_4_ ferrite calcined at 800 °C, the average values of growth inhibition were 58.3%, 54.0%, and 51.7%, respectively. From the dispersion control, it was observed that there was no influence on the antibacterial action of the ferrite.

Table 2 presents the values obtained for IC50 for the inhibition analyzes of *S. aureus* strains. Thus, MIC was considered to be greater than 1 mg/mL, with only the IC50 values expressed in the table. This varied from 0.46 to 1 mg/mL for the three incubation times tested. The calcination temperature did not induce any significant effect, however, for the ferrites ZnFe_2_O_4_ and Zn_0.5_Co_0.5_Fe_2_O_4_ calcined at 250 °C, they showed better results. However, amorphous particles probably have superior inhibitory effects when compared to crystalline NP.

**Table 2:** Table referring to the IC50 values found for tests with ferrites in contact with *S. aureus* strains at different incubation times. *n* = 3

IC50 obtained for strains of *S. aureus*			
Ferrite	Incubation time		
	24 h	48 h	72 h
ZnFe_2_O_4_ at 250 °C	460.7	480.1	464.7
ZnFe_2_O_4_ at 800 °C	522.4	547.6	521.2
CoFe_2_O_4_ at 250 °C	948.4	>1000	>1000
CoFe_2_O_4_ at 800 °C	>1000	>1000	>1000
Zn_0.5_Co_0.5_Fe_2_O_4_ at 250 °C	465.0	549.6	949.1
Zn_0.5_Co_0.5_Fe_2_O_4_ at 800 °C	473.6	515.9	966.9

Figure 8 expresses the inhibition growth for the ferrites calcined at 250 °C (800 °C) of ZnFe_2_O_4_, CoFe_2_O_4_, and Zn_0.5_Co_0.5_Fe_2_O_4_ in contact with *E. coli* strains (*n* = 3) in the three incubation times evaluated. For the ZnFe_2_O_4_ ferrite calcined at 250 °C, the inhibition averages in the 3 incubation times, were equal to 54.7%, 46.4%, and 40.8%, respectively. And the average values obtained for the ZnFe_2_O_4_ ferrite calcined at 800 °C were equal to 51.0%, 44.6%, and 38.1%, respectively. For the CoFe_2_O_4_ ferrite calcined at 250 °C, in the incubation times of 24, 48, and 72 h, the percentage values of inhibition were equal to 38.5%, 31.4%, and 26.8%, respectively. And for the CoFe_2_O_4_ ferrite calcined at 800 °C, the percentage inhibition values were 34.3%, 28.4%, and 25.1%. For the Zn_0.5_Co_0.5_Fe_2_O_4_ ferrite calcined at 250 °C, the mean percentage inhibition values were equal to 48.6%, 42.8%, and 38.0%, respectively. And for Zn_0.5_Co_0.5_Fe_2_O_4_ ferrite, the mean percentage inhibition values were 44.8%, 38.2% and 35.5%, respectively.

Table 3 presents the IC50 values for the analysis of inhibition of *E. coli* strains obtained by non-linear regression. At all incubation times, and both calcination temperatures, MIC values were greater than 1 mg/mL. The IC50 value ranged from 0.91 to 1 mg/mL for the three incubation times tested and the two calcination temperatures.

**Table 3:** Table referring to the IC50 values found for tests with ferrites in contact with E. coli strains. *n* = 3

IC50 obtained for strains of *E. coli*			
Ferrite	Incubation time		
	24 h	48 h	72 h
ZnFe_2_O_4_ at 250 °C	913.8	>1000	>1000
ZnFe_2_O_4_ at 800 °C	980.2	>1000	>1000
CoFe_2_O_4_ at 250 °C	>1000	>1000	>1000
CoFe_2_O_4_ at 800 °C	>1000	>1000	>1000
Zn_0.5_Co_0.5_Fe_2_O_4_ at 250 °C	>1000	>1000	>1000
Zn_0.5_Co_0.5_Fe_2_O_4_ at 800 °C	>1000	>1000	>1000

A study carried out with Fe_3_O_4_ NPs in contact with the resistant species *E. coli* showed that the antibacterial effect promoted by ferrite in this bacterium is dependent on the concentration. In addition, the authors reported that the size of the ferrite was related to the antibacterial action [41]. A study carried out with zinc oxide, iron oxide, and Zn/Fe oxide, showed greater inhibitory action for NPs with a higher molar zinc concentration, obtaining greater inhibition for strains of *S. aureus* than for strains of *E. coli* [20, 42, 43] performed an antibacterial evaluation with Zn_0.5_Co_0.5_Fe_2_O_4_ ferrite in MRSA (*S. aureus* Methicillin Resistant) and *E. coli* and were able to confirm greater inhibitory action for *S. aureus*. This inhibition decreased according to the incubation time, in 2 h there was an approximately 80% inhibition for *S. aureus* and 70% for *E. coli* and in 24 it decreased to about 65% and 50% inhibition.

**Fig. 9:**
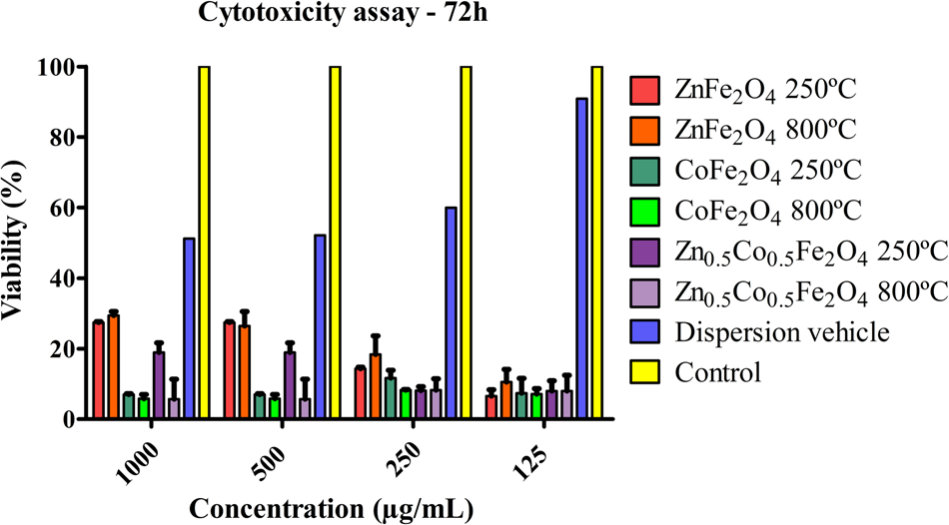
Vero cells were incubated with various concentrations of ZnFe_2_O_4_, CoFe_2_O_4_, and Zn_0.5_Co_0.5_Fe_2_O_4_ NPs (125-1000 µg/mL) for 72 h and viability cell was measured using MTT assay. The results are expressed as the mean + standard derivation of triplicate experiment. Differences between the dispersion component and nanoparticles were measured using ANOVA test. The results showed statistical significance between the NPs and the dispersion component (*p* < 0.005)

The cytotoxicity of NPs on Vero cells was evaluated by MTT assay. Figure 9 shows the viability of Vero cells exposed to 125, 250, 500, and 1000 μg/mL of ZnFe_2_O_4_ NPs, CoFe_2_O_4_ NPs, and Zn_0.5_Co_0.5_Fe_2_O_4_ NPs. The specific values were IC_50_ = 16.72 µg/mL for ZnFe_2_O_4_ NPs calcined at 250 °C, IC_50_ = 16.28 µg/mL for ZnFe_2_O_4_ NPs calcined at 800 °C, IC_50_ = 17.65 µg/mL for CoFe_2_O_4_ NPs calcined at 250 °C, IC_50_ = 17.73 µg/mL for CoFe_2_O_4_ NPs calcined at 800 °C, IC_50_ = 13.42 µg/mL for Zn_0.5_Co_0.5_Fe_2_O NPs calcined at 250 °C and IC_50_ = 17.24 µg/mL for Zn_0.5_Co_0.5_Fe_2_O NPs calcined at 800 °C. The value IC_50_ = 120.5 µg/mL for dispersion vehicle (propylene glycol).

#### Agar diffusion test

**Fig. 10:**
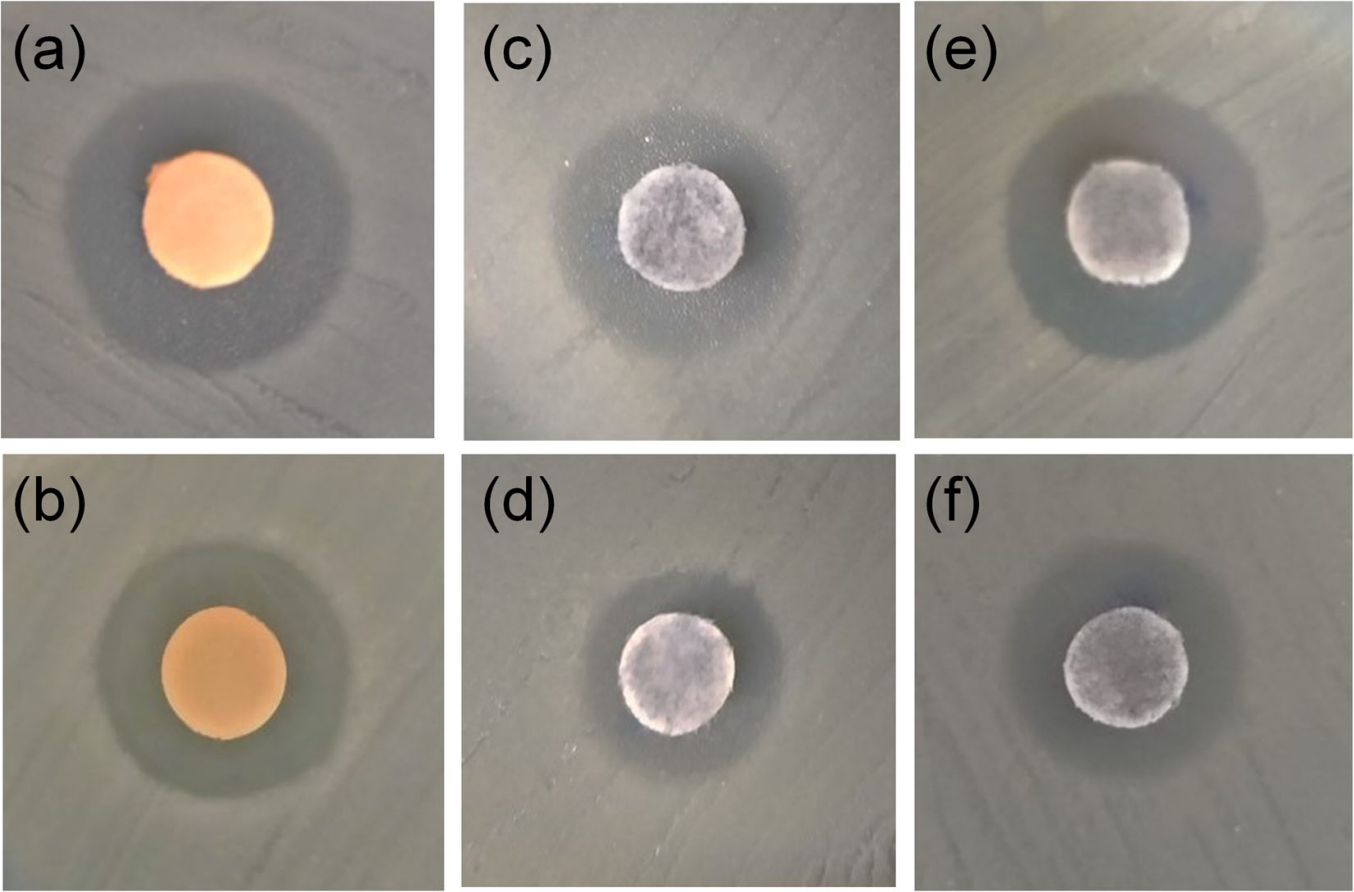
Disc diffusion test for *S. aureus* strains (*n* = 3) and inhibition halos formed by ferrites dispersed by nanoemulsion. ZnFe_2_O_4_ calcined at 250 °C (**a**), and 800 °C (**b**). CoFe_2_O_4_ calcined at 250 °C (**c**), and 800 °C (**d**). Zn_0.5_Co_0.5_Fe_2_O_4_ calcined at 250 °C (**e**), and 800 °C (**f**)

Results on the antimicrobial activity of CoFe_2_O_4_, ZnFe_2_O_4_, and Zn_0.5_Co_0.5_Fe_2_O_4_ NPs are rare in the literature. In this work, the antimicrobial activities of the synthesized NPs were tested against Gram-positive and Gram-negative bacteria. The antimicrobial effects of the NPs were qualitatively measured by performing agar diffusion test against all the test microorganisms. The results of zones of inhibition are shown in Table 4. The agar plate inhibition tests were performed by the disc diffusion method, as a way to confirm the inhibitory action of the ferrites prepared here. The disk inhibition tests, following the Kirby–Bauer method [44]. The absence of microbial growth around the NPs is an indirect measure of the ability of the NP to inhibit the growth. With a concentration of 2 mg/mL, was evident the formation of growth inhibition halos around the disks on the medium with the bacterium. After the dispersion using the nanoemulsion was possible to obtain clearer and evident inhibition halos for all synthesized ferrites, as shown in Fig. 10.

**Table 4:** Averages of the diameters of the inhibition halos of each ferrite tested in strains of *S. aureus* (*n* = 3) and *E. coli* (*n* = 3)

Ferrite	Average halo diameters (mm) for each species	
	*S. aureus*	*E. coli*
ZnFe_2_O_4_ at 250 °C	13.1	8.8
ZnFe_2_O_4_ at 800 °C	12.1	7.8
CoFe_2_O_4_ at 250 °C	9.4	6.9
CoFe_2_O_4_ at 800 °C	8.7	6.0
Zn_0.5_Co_0.5_Fe_2_O_4_ at 250 °C	10.9	7.7
Zn_0.5_Co_0.5_Fe_2_O_4_ at 800 °C	10.5	7.0
Chloramphenicol	24	20
Negative control	0	0
Dispersion control	0.9	0.6

Figure 10 shows a visual difference between the formed diameters by the inhibition halos created by zinc, cobalt ferrites, and Co^2+^ substituted zinc ferrite calcined at 250 °C and 800 °C in contact with *S. aureus* strains. It was observed that the calcination temperature changed the diameter of the formed halo.

Figure 10a, b shows the halos formed for ZnFe_2_O_4_ NPs calcined at 250 and 800 °C when in contact with *S. aureus* strains. The diameter measurement of the formed halo for ZnFe_2_O_4_ NPs calcined at 250 and 800 °C were 13.1 and 12.1 mm, as shown in Table 4.

Figure 10c, d shows the halos formed for CoFe_2_O_4_ NPs calcined at 250 and 800 °C when in contact with *S. aureus* strains. The diameter measurement of the formed halo for CoFe_2_O_4_ NPs calcined at 250 and 800 °C was 9.4 mm and 8.7 mm, as shown in Table 4.

The last NP placed in contact with *S. aureus* strains in the Agar diffusion test was the Zn_0.5_Co_0.5_Fe_2_O_4_, as shown in Fig. 10e, f. In this case, the diameter of the halo formed did not change significantly. The diameter measurement of the formed halo for Zn_0.5_Co_0.5_Fe_2_O_4_ NPs calcined at 250 and 800 °C were 10.9 mm and 10.5 mm, as shown in Table 4. In general, the diameters of the halos formed for ZnFe_2_O_4_ NPs were slightly larger for NPs calcined at low temperature. These measured results suggest that the crystalline structure of NPs calcined at low temperature was slightly more effective in inhibiting *S. aureus*. XRD data shows that at low temperature, calcined NPs have more characteristics of amorphous material.

The second step was to place the same NPs in the agar diffusion test in contact with *E. coli* strains. The procedure used was the same as for *S. aureus* strain.

**Fig. 11:**
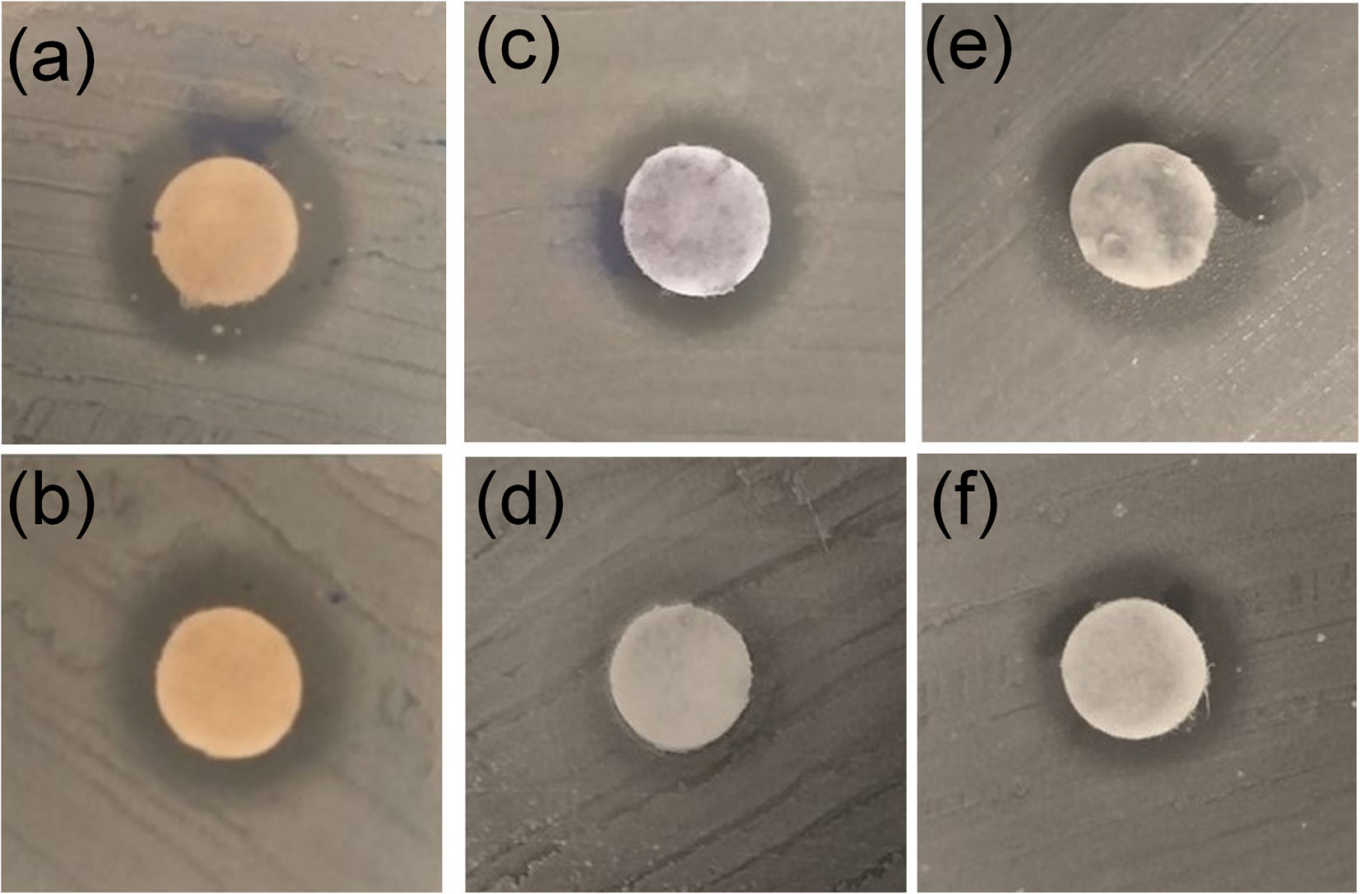
Disc diffusion test for *E. coli* strains (*n* = 3) and inhibition halos formed by ferrites dispersed by nanoemulsion. ZnFe_2_O_4_ calcined at 250 °C (**a**), and 800 °C (**b**). CoFe_2_O_4_ calcined at 250 °C (**c**), and 800 °C (**d**). Zn_0.5_Co_0.5_Fe_2_O_4_ calcined at 250 °C (**e**), and 800 °C (**f**)

Figure 11a, b shows the halos formed for ZnFe_2_O_4_ NPs calcined at 250 and 800 °C when in contact with *E. coli* strains. The diameter measurement of the formed halo for ZnFe_2_O_4_ NPs calcined at 250 and 800 °C was 8.8 mm and 7.8 mm, as shown in Table 4.

Figure 11c, d shows the halos formed for CoFe_2_O_4_ NPs calcined at 250 and 800 °C when in contact with *E. coli* strains. The diameter measurement of the formed halo for CoFe_2_O_4_ NPs calcined at 250 °C and 800 °C were 6.9 mm and 6.0, as shown in Table 4.

The last NP placed in contact with *E. coli* strains in the Agar diffusion test was the Zn_0.5_Co_0.5_Fe_2_O_4_, as shown in Fig. 11e, f. The diameter measurement of the formed halo for Zn_0.5_Co_0.5_Fe_2_O_4_ NPs calcined at 250 and 800 °C was 7.7 mm and 7.0 mm, as shown in Table 4. Similarly, the diameters of the halos formed for ZnFe_2_O_4_ NPs were slightly larger for NPs calcined at low temperature. These measured results suggest that the crystalline structure of NPs calcined at low temperature was slightly more effective in inhibiting *E. coli* strains.

**Fig. 12:**
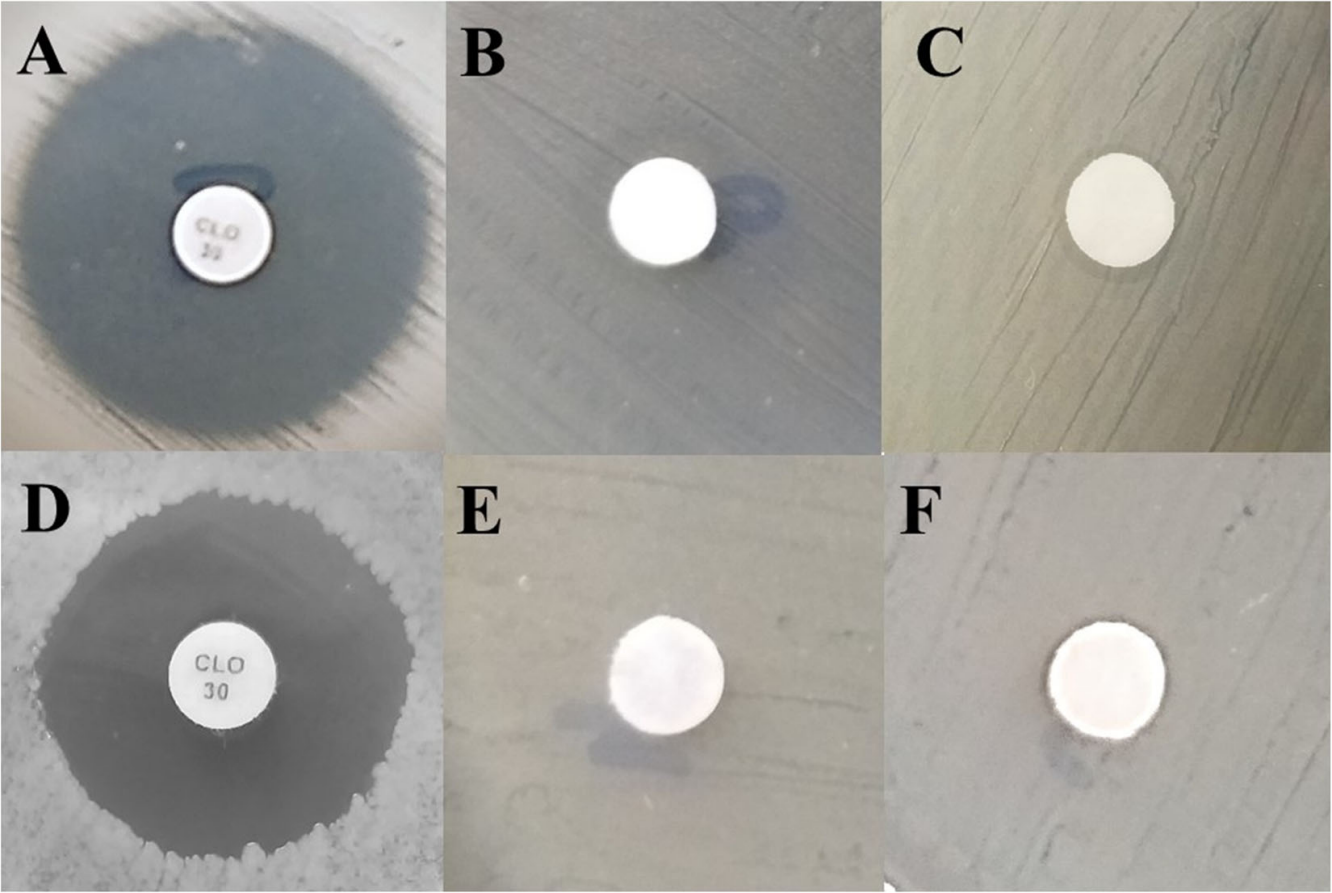
Disc diffusion test for strains of *S. aureus* (**A**–**C**) and *E. coli* (**D**–**F**), *n* = 3, and inhibition halos formed by the controls. **A**, **D** Chloramphenicol; **B**, **E** negative control (distilled water); and (**C**, **F**) dispersion control (nanoemulsion)

The validity of the agar diffusion method was verified using the classic antibiotic chlorophenicolc. It is used as a controls. Figure 12 shows the inhibition halos for bacteria *S. aureus* in contact with chloramphenicol antibiotic controls (A), negative control (distilled water in the disc) (B), and control of the dispersion used in the test (nanoemulsion deposited in the disc) (C). Was also compared the antibiotic chloramphenicol effect (D), negative control (E), and the nanoemulsion (F) in contact with the *E. coli* bacteria.

The results presented in the figures show that the nanoemulsion did not interfere significantly in the results of inhibition of the produced NPs, when tested with *E. coli* and *S. aureus*, as shown in Table 4. A study carried out with α-Fe_2_O_3_ (hematite) also showed that *S. aureus* bacteria are more sensitive than *E. coli* in an agar diffusion test, which is due to the greater resistance of Gram-negative because it has an extra lipid membrane, which makes it difficult the passage of NPs [45]. The authors Mandal et al. [46], showed that the inhibition halos formed by zinc ferrites are larger for *S. aureus* than when testing with *E. coli*. In this study, an inhibition zone equal to 22 mm was obtained for *S. aureus* and 14.6 mm for *E. coli*. In general, our results for the sizes of inhibition halos are of the same order of magnitude as those obtained by Mandal et al. Another study whose results were similar to ours is with NPs of Mn_1−*x*_Ni_*x*_Fe_2_O_4_, the authors obtained, using the agar well diffusion method, halos equal to (18 mm) for *S. aureus* and smaller halos (12 mm) for *E. coli* [16]. Tests using the modified Kirby–Bauer diffusion method showed that for greater zinc molarities in the mixed cobalt and zinc ferrite, greater inhibition halos were formed [24]. Similar to this study, it was also observed in the present study that the halos formed for the cobalt ferrites were inferior to the halos formed for the other ferrites. Ashour et al. [47], in tests with Zn_0.5_Co_0.5_Fe_2_O_4_ by disk diffusion, showed that strains of *S. aureus* compared to strains of *E. coli* showed a greater inhibition halo, which was 16 mm for positive-Gram and 11 mm for negative-Gram. Ashour et al. [47], which tested *S. aureus*, in contact with mixed zinc and cobalt ferrite (Zn_0.75_Co_0.25_Fe_2_O_4_), in which a high inhibitory power was demonstrated, with the formation of 15 mm inhibition halos. Sharma et al. [48], using cobalt ferrite to combat *S. aureus*, *E. coli*, and other strains, showed that the tested ferrite showed inhibition potential for both classes of bacteria, as both are negatively charged, which favor interaction electrostatic between the cell and the NPs or ions released from them.

Some factors, such as the composition and structure of the cell wall, may also be related to the rate of bacterial death. It is seen that the inhibitory capacity for *S. aureus* and *E. coli* bacteria are different. However, the structure of the bacteria can promote greater attraction to NPs, since *E. coli* consists of lipid A, lipopolysaccharide, and peptidoglycan; while the cell wall of *S. aureus* is composed mostly of peptidoglycan [23, 49–51]. Some studies suggest that the inhibitory action generated by NPs is due to the generation of reactive oxygen species (ROS), which end up causing bacterial death. Results by Arakha et al. [52] showed that the antimicrobial mechanism involved in bacterial death is through the release of ROS when in contact with iron oxide NPs, in which NPs with positive and negative surface charges were tested, obtaining greater antimicrobial activity to those positively charged. The mechanism generated by photocatalytic reactions causes oxidative stress in bacteria. These end up suffering a rupture of the cell membrane with the leakage of cytoplasmic materials [53]. Other possible mechanisms are the attraction of NPs to the bacterial surface, due to the differences in loads. Metal cations are attracted by the negative surface charge of bacteria, they accumulate allowing entry into the cell, causing pores in the wall/membrane and loss of intracellular content [54].

## Conclusions

The synthesis of ZnFe_2_O_4_, CoFe_2_O_4_, and Zn_0.5_Co_0.5_Fe_2_O_4_ ferrites was successful by the sol-gel method. FTIR measurements showed important peaks for the transition metal ferrites of Zn and Co. The analysis by XRF detected higher percentages of iron with proportions of 2:1 for both Fe:Zn and Fe:Co. X-ray diffraction made it possible to structurally characterize ferrites, confirming that the material is of the crystalline type and that the degree of crystallinity has increased for higher calcination temperatures (800 °C). The micrographs obtained by transmission electron microscopy showed that the ferrites calcined at a lower temperature, 250 °C were small in size compared to the ferrites calcined at a higher temperature, 800 °C. The antibacterial activity of the three ferrites was partially shown for the tests at concentrations of 1 mg/mL, 0.5 mg/mL, and 0.25 mg/mL with MIC greater than 1000 µg/mL for the two bacteria tested. According to the halos obtained, for discs at a concentration of 2 mg/mL, it was concluded that there was greater inhibition efficiency in the order of ZnFe_2_O_4_ > Zn_0.5_Co_0.5_Fe_2_O_4_ > CoFe_2_O_4_. Thus, it is concluded that the synthesized ferrites have dose-inhibitory capacity dependence.
